# Glucose-Regulated Protein 78-Induced Myeloid Antigen-Presenting Cells Maintained Tolerogenic Signature upon LPS Stimulation

**DOI:** 10.3389/fimmu.2016.00552

**Published:** 2016-12-01

**Authors:** Muyang Yang, Fan Zhang, Kai Qin, Min Wu, Heli Li, Huifen Zhu, Qin Ning, Ping Lei, Guanxin Shen

**Affiliations:** ^1^Department of Immunology, School of Basic Medicine, Tongji Medical College, Huazhong University of Science and Technology, Wuhan, China; ^2^Department of Infectious Disease, Institute of Infectious Disease, Tongji Hospital of Tongji Medical College, Huazhong University of Science and Technology, Wuhan, China

**Keywords:** glucose-regulated protein 78, antigen-presenting cells, regulatory T cell, immune regulation, autoimmune disease

## Abstract

The 78-kDa glucose-regulated protein (Grp78) is stress-inducible chaperone that mostly reside in the endoplasmic reticulum. Grp78 has been described to be released at times of cellular stress and as having extracellular properties that are anti-inflammatory or favor the resolution of inflammation. As antigen-presenting cells (APCs) play a critical role in both the priming of adaptive immune responses and the induction of self-tolerance, herein, we investigated the effect of Grp78 on the maturation of murine myeloid APCs (CD11c^+^ cells). Results showed that CD11c^+^ cells could be bound by AF488-labeled Grp78 and that Grp78 treatment induced a tolerogenic phenotype comparable to immature cells. Furthermore, when exposed to lipopolysaccharide, Grp78-treated CD11c^+^ cells (DC_Grp78_) did not adopt a mature dendritic cell phenotype. DC_Grp78_-primed T cells exhibited reduced proliferation along with a concomitant expansion of CD4^+^CD25^+^FoxP3^+^ cells in pancreaticoduodenal lymph nodes and induction of T cell apoptosis *in vitro* and *ex vivo*. The above work suggests that Grp78 is an immunomodulatory molecule that could aid resolution of inflammation. It may thus contribute to induce durable tolerance to be of potential therapeutic benefit in transplanted allogeneic grafts and autoimmune diseases such as type I diabetes.

## Introduction

The endoplasmic reticulum (ER) chaperone Grp78/BiP is a central regulator of ER homeostasis, and its upregulation is widely used as a sentinel marker under pathologic conditions for ER stress, such as hypoxia, hypoglycemia, infection, nutrient deprivation, low-calcium, and others (1, 2). As an important cellular defense mechanism, Grp78 is highly increased in tumors and process to facilitate cell invasion, growth, and metastasis (3–6). Upregulation of Grp78 could lead to the translocation of Grp78 to other cellular locations and secretion of Grp78 into the extracellular compartment (7–11).

Our previous work confirmed that overexpression of Grp78 in insulinoma cells prolonged cell survival and subsequently maintained normoglycemia in diabetic Balb/c mice (12, 13). Interestingly, in the following experiments, it was found that Grp78-overexpressing insulinoma cell-treated animals secreted high levels of IL-4, suggesting the immunosuppressive ability of Grp78 in beta cell transplantation (13). However, it was not clear how Grp78 exerted such immunomodulatory effect for improvement of alloimmunity.

Corrigall et al. reported that recombinant human Grp78 (rhGrp78) could prevent the induction of experimental arthritis when it was given i.v. before the induction of CIA (14) or during active disease to induce permanent remission of inflammation in CIA (15). At that time, they postulated that Grp78 just acted as an autoantigen to prevent the induction of arthritis. However, results suggested that Grp78 might have immunomodulatory properties because it appeared to be able to significantly suppress IgG2 and IgG1 anti-collagen Abs. Later, they confirmed that rhGrp78 affected the differentiation of human peripheral blood monocytes (PBMO) into dendritic cells (DCs) and reduced their expressions of HLA-DR and CD86, and thence the development of regulatory T cells (16). Furthermore, in 2011, Grp78 was proposed as one of the founding members of resolution-associated molecular patterns (RAMPs) family that are anti-inflammatory and favor the resolution of inflammation (9).

We previously reported that recombinant mouse Grp78 (rmGrp78) could program splenic B cells into CD19^hi^FasL^+^/PD-L1^+^ IL-10-producing B cells to suppress T cell proliferation (17). Besides regulatory B cells, it is valuable to investigate whether Grp78 could program other murine antigen-presenting cells (APCs) into tolerogenic cells to prevent unwanted immune reactions in mice. In this study, we assessed the effect of rmGrp78 on DCs, which have a role in both the priming of adaptive immune responses and the induction of self-tolerance (18–20). Results showed that Grp78 could induce myeloid APCs (CD11c^+^ cells) to differentiate into a distinct tolerogenic cells, and these cells could retain tolerogenic signature after lipopolysaccharide (LPS) stimulation. DC_Grp78_-primed T cells exhibited reduced proliferation along with a concomitant expansion of Tregs and induction of T cell apoptosis *in vitro* and *ex vivo*. The above work suggests that Grp78 can contribute to induce durable tolerance to be of potential therapeutic benefit in transplanted allogeneic grafts and autoimmune diseases such as type I diabetes.

## Materials and Methods

### Preparation of Recombinant Murine Grp78

Recombinant murine Grp78 was prepared as our previous report (17). Briefly, plasmid encoding full length of murine Grp78 was transformed into *Escherichia coli* BL21 to generate Grp78. Protein was purified using GST Fusion Protein Purification Kit (GenScript, USA) and identified by SDS-PAGE and immunoblotting. Endotoxins were removed by the Toxin Eraser™ Endotoxin Removal Kit (GenScript), and the endotoxin contamination was less than 1 EU/mg protein. The Grp78 concentration was detected by the BCA Protein Assay Kit (Beyotime, Beijing, China). Control extracts from empty vector-transformed *E. coli* BL21 were prepared in the same way.

### Animals

The female BALB/c mice (HFK Bioscience Co., Ltd., Beijing, China) used were between 6 and 8 weeks of age. Experiments were approved by the Ethics Committee of Tongji Medical College of Huazhong University of Science and Technology.

### Cell Cultures

Bone marrow (BM)-derived CD11c^+^ cells from normal BALB/c mice were generated as described previously (21). Cells (80% purity, 90% viability) were harvested after 7 days of culture with 10 ng/ml GM-CSF, 10 ng/ml IL-4, 10 μg/ml Grp78 or control extracts, and named as DC_Grp78_ or immature DC accordingly. LPS (500 ng/ml, Sigma, St. Louis, MO, USA) was added for the final 18–24 h to generate DC_Grp78+LPS_ or DC_LPS_. Phenotypic characteristics were determined after 7 days of culture. Medium was replaced by fresh warmed medium with GM-CSF, IL-4, and Grp78. Cytokines and Grp78 were maintained at that concentration throughout medium changes.

Insulinoma cell line NIT (10^7^) in the logarithmic growth phase were heated at 37°C for 30 min, washed in PBS, and subjected to four freeze (liquid nitrogen) and thaw (37°C water bath) cycles to obtain crude lysates. Prepared NIT lysates were added at 100 μg/10^6^ DCs and incubated overnight to obtain NIT lysate-pulsed DCs.

CD4^+^CD25^−^ T cells and CD8^+^ T cells were sorted by MACS (Miltenyi Biotec, Germany) from splenocytes. They were mixed with CD11c^+^ cells, respectively, in 96-well round-bottom plates with 0.3 μg/ml anti-CD3 molecular complex (BD) stimulation of T lymphocytes for 72 h. Proliferation of T cells was measured by CFSE (5–10 μM, Invitrogen) staining or incorporation of [methyl-^3^H]thymidine (1 μCi/well, BioCreater Co., Wuhan, China) for 6 h. Apoptosis was detected by 7-AAD staining. To analyze the differentiation of CD11c^+^ cells pulsed T cell, the mixed cells were transferred into 96-well flat-bottom plates for further 3-day culture.

### Identification of Binding of Grp78 with CD11c^+^ Cells

Grp78 was labeled with AF488 dye using Alexa Fluor^®^ 488 Protein Labeling Kit (Invitrogen, Eugene, OR, USA) as per the manufacturer’s recommendation. Protein conjugates were purified using Protein Labeling Kit (Life Technologies, USA). BSA was labeled as a negative control. CD11c^+^ cells were harvested at different time (0/2/4/6 days after derived from BM) and cytospined and fixed with 4% paraformaldehyde and stained with Grp78-AF488 at 4 or 37°C for 1 h. Confocal microscope (Olympus FV500, Tokyo, Japan) was used to visualize the binding of Grp78 with cells. Same amount of BSA-AF488 was used as negative control.

### Reagents for Flow Cytometric Analysis

The following fluorescein-labeled antibodies were used for cell surface marker analysis: CD11c-PE-cy7, CD40-APC, CD83-APC, CD80-PE-cy5, MHC-II-FITC, B7-H3-PE, B7-H4-PE, CD3-PE, CD8a-PE-Cy5, CD4-APC, CD25-PE-Cy7, and Foxp3-FITC. They were purchased for eBioscience (San Diego, CA, USA) except for CD83-APC from BioLegend (USA). For intracellular staining, Via-probe was added prior to fixation and permeabilization. Data were acquired with flow cytometer and analyzed using FlowJo software.

### Cytokine Analysis

ELISA kits were used for detection of IL-10, TGF-β, TNF-α, HMGB-1, and IFN-γ in cell culture supernatants (BOSTERBIO, Wuhan, China). Nitrite was measured as representative of NO synthesis in DCs’ culture supernatants using the Griess reagent (Sigma-Aldrich) and measuring absorbance at 540 nm.

### Relative Quantitative Real-time PCR

The total RNA was isolated from the CD11c^+^ cells using the Iso-plus reagent (TaKaRa, Dalian, China) according to the manufacturer’s protocol. cDNA was synthesized using M-MLV reverse transcriptase (TaKaRa). The real-time PCR reactions were performed in duplicate using a Light Cycler 480 PCR system (Roche, IN, USA) following the manufacturer’s instructions. Data were normalized using the geometric mean of housekeeping gene GAPDH. The primer sets were shown in Table 1.

**Table 1:** **Sequence of specific primers used**.

Name	Primer sequence
GAPDH	Sense: 5′-CCCCTTCATTGACCTCAACTAC-3′
	Anti-sense: 5′-CTCGCTCCTGGAAGATGGTGAT-3′
IDO1	Sense: 5′-CTGCCTCCTATTCTGTCTTATGC-3′
	Anti-sense: 5′-CTTTCAGGTCTTGACGCTCTACT-3′
Arg-1	Sense: 5′-GGGAAGACAGCAGAGGAGGTG-3′
	Anti-sense: 5′-AGGTAGTCAGTCCCTGGCTTAT-3′
Cox-2	Sense: 5′-GATAACCGAGTCGTTCTGCCAAT-3′
	Anti-sense: 5′-CCTGGTCGGTTTGATGTTACTG-3′
NOS2	Sense: 5′-CAGGGAATCTTGGAGCGAGTTG-3′
	Anti-sense: 5′-GTGAGGGCTTGGCTGAGTGAG-3′
TGF-β1	Sense: 5′-GACCGCAACAACGCCATCTAT-3′
	Anti-sense: 5′-GACAGCCACTCAGGCGTATCAG-3′
IFN-γ	Sense: 5′-CTCTGAGACAATGAACGCTACAC-3′
	Anti-sense: 5′-CCTTTTGCCAGTTCCTCCAGATAT-3′
TNF-α	Sense: 5′-GCAGGTCTACTTTGGAGTCATTG-3′
	Anti-sense: 5′-CAGGTCACTGTCCCAGCATCT-3′
IL-17A	Sense: 5′-CCTCAGACTACCTCAACCGTTC-3′
	Anti-sense: 5′-CTCTTCAGGACCAGGATCTCTT-3′
IL-4	Sense: 5′-GAGCCATATCCACGGATGCGAC-3′
	Anti-sense: 5′-CGAAGCACCTTGGAAGCCCTAC-3′
IL-6	Sense: 5′-GACTTCCATCCAGTTGCCTTCT-3′
	Anti-sense: 5′-TCTCATTTCCACGATTTCCCAG-3′
IL-10	Sense: 5′-GGACAACATACTGCTAACCGAC-3′
	Anti-sense: 5′-CATGGCCTTGTAGACACCTTG-3′
IL-12a	Sense: 5′-GGTCAGCGTTCCAACAGCCTC-3′
	Anti-sense: 5′-ATGTGCTGGTTTGGTCCCGTGT-3′

### DC Adoptive Transfer

MACS (Miltenyi Biotec) sorted CD11c^+^ DCs (5 × 10^6^) were injected intraperitoneally into 6- to 8-week-old BALB/c mice every 7 days for three times. Mice were sacrificed 1 week after final injection, and pancreaticoduodenal lymph nodes (PLNs), blood, and spleen were dissected.

### Statistical Analysis

Statistical significance was determined by *T*-test, and percentage data were analyzed by Mann–Whitney *U* test using SPSS 17.0 statistical software (SPSS Inc., USA). All values were expressed as means ±SD. A *P* value of < 0.05 was considered significant, and error bars were presented in SEM.

## Results

### Grp78 Could Be Bound By CD11c^+^ Cells

**Figure 1:**
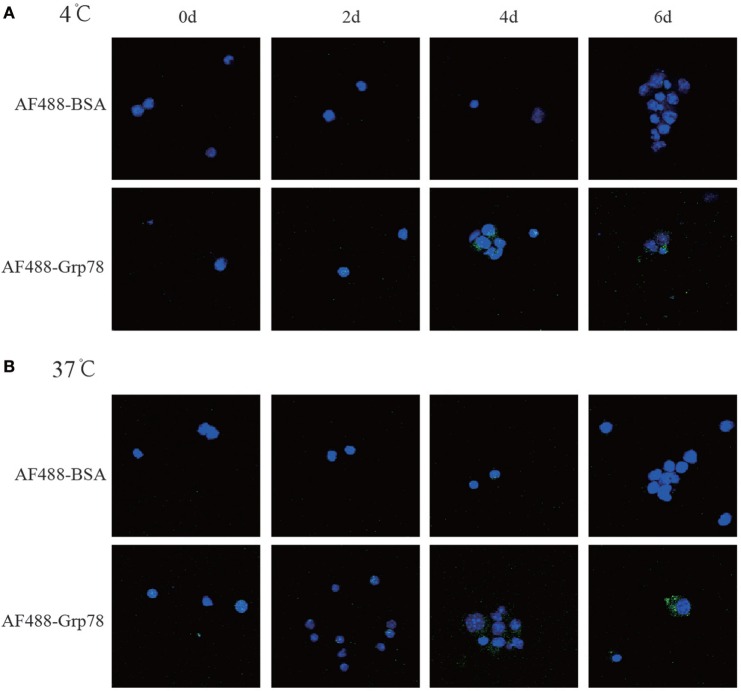
**Grp78 bound with CD11c^+^ cells**. CD11c^+^ cells at different days of culture were cytospined for 1 min at 1000 rpm and then cocultured with AF488-labeled Grp78 (1 μmol/ml) at 4°C **(A)** or 37°C **(B)** for 1 h. Cell nuclei were stained with DAPI. Confocal microscope was used to check the Grp78^+^ cells. Same amount of AF488-BSA was used as a negative control.

To investigate the effect of Grp78 on CD11c^+^ cells, it was essential to check whether Grp78 could be bound by cells. Confocal imaging studies showed that nearly all CD11c^+^ cells at 4- and 6-day culture could be stained with Grp78-AF488, but none with BSA-AF488 (Figure 1). And the location of AF488-Grp78 in cells showed no difference between 4 and 37°C. These figures confirmed the binding of Grp78 with CD11c^+^ cells.

### Grp78 Induced an Anti-inflammatory Cell Phenotype

**Figure 2:**
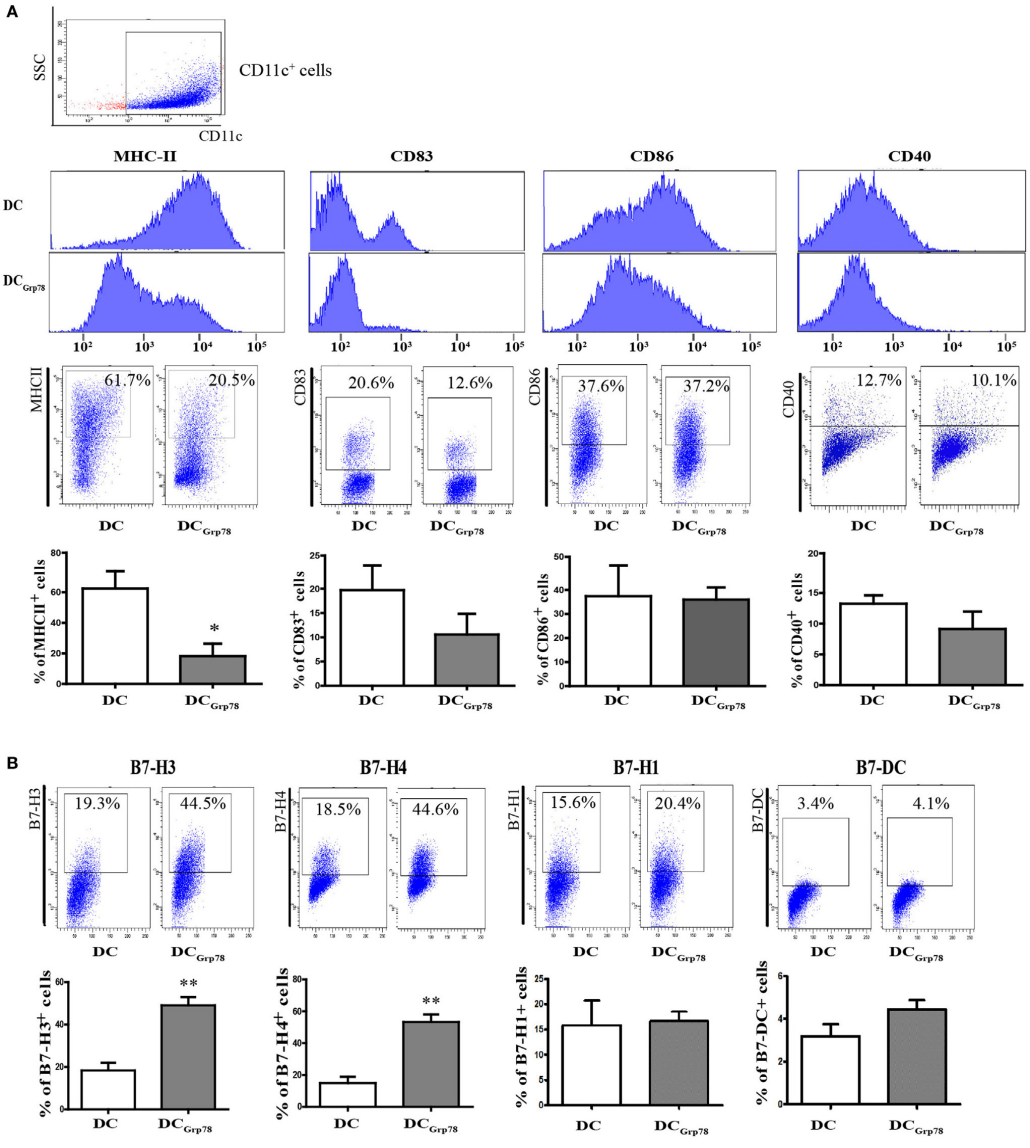

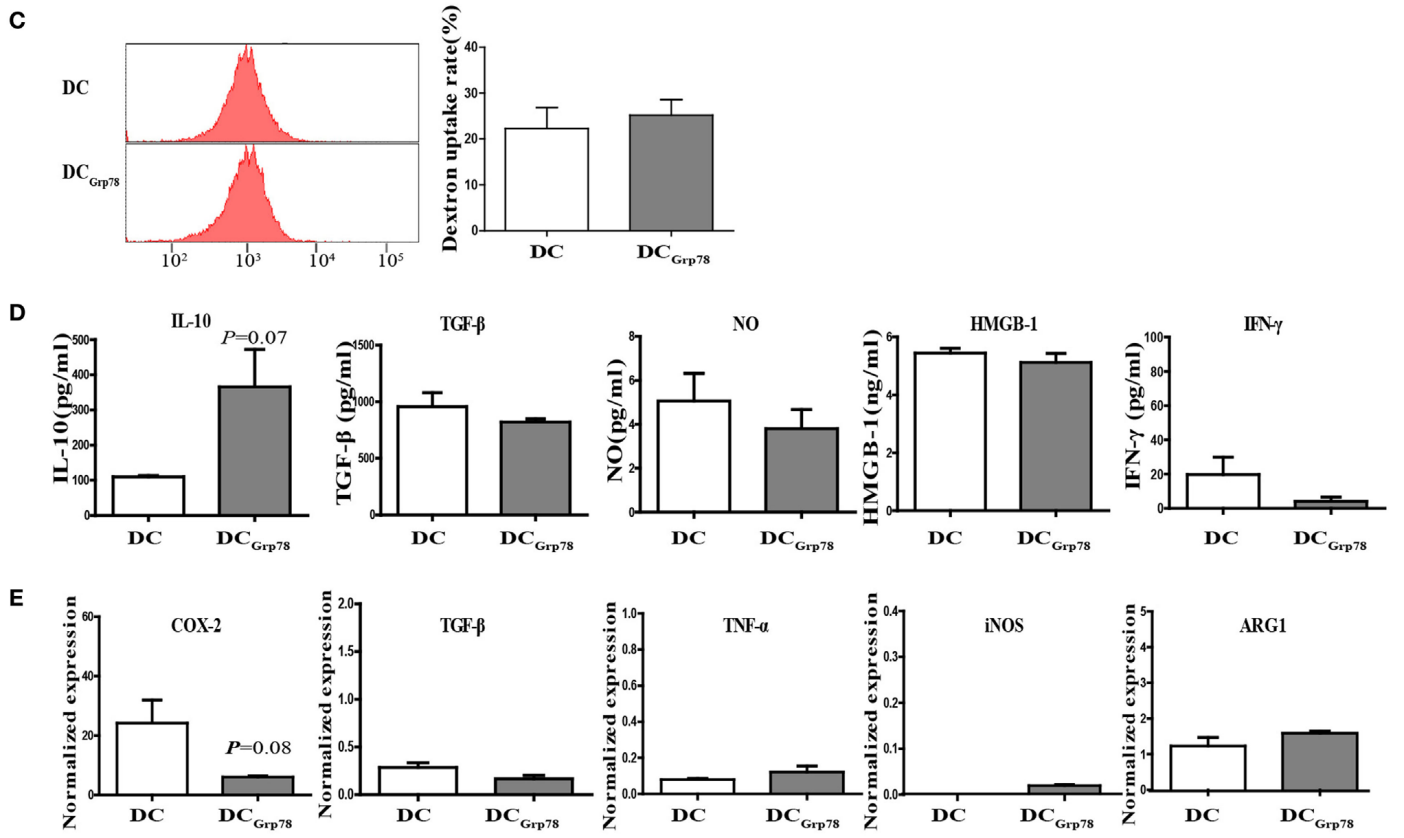
**Grp78 induced a distinct anti-inflammatory phenotype**. Bone marrow cells (1 × 10^6^) were costimulated for 7 days with IL-4, GM-CSF, and with/without Grp78. **(A)** Flow cytometry analyses of MHC-II, CD83, CD80, and CD40 expression on CD11c^+^ cells. Mean fluorescence intensity (upper), representative flow cytometric dot plots (middle), and percentage of positive cells (lower) were depicted. **(B)** Percentage of B7-H3/B7-H4/B7-1/B7-DC cells in CD11c^+^ cell population. **(C)** Endocytosis of CD11c^+^ cells assessed by 1 h incubation with dextran-rhodamine. MFI (left) and percentage of rhodamine positive cells (right) were depicted. **(D)** Cell culture supernatants were analyzed for IL-10, TGF-β, NO, HMGB-1, and IFN-γ by ELISA. **(E)** Gene expression of COX-2, TGF-β, TNF-α, iNOS, and ARG-1 assessed by qPCR. All results were representative of three independent experiments. Statistical comparisons were conducted against untreated controls. Error bars were presented in SEM. *P < 0.05 vs. DC, **P < 0.01 vs. DC.

To assess the effect of Grp78 on modulation of CD11c^+^ cells, cells were cultured in the presence of Grp78 for 7 days and then subjected to phenotype analysis. Addition of Grp78 had downregulatory effects on the expression of MHC-II and DC maturation markers CD83 on DC_Grp78_ cells compared with the immature cells (Figure 2A), diminishing their critical antigen presentation function. Among these molecules, MHC-II showed significant decrease not only in the mean intensity but also in the percentage in CD11c^+^ cells. Consistent with the downregulation of costimulatory molecules, DC_Grp78_ cells showed increased frequencies of B7-H3 and B7-H4 co-inhibitory molecules, but not B7-H1 and B7-DC (Figure 2B). Endocytosis is another important function for APCs during inflammation for clearance of both apoptotic cells and cellular debris from the local environment. The endocytic potential of CD11c^+^ cells was assessed by the uptake of dextran-rhodamine. However, DC_Grp78_ and immature DCs showed no evident differences in endocytic activity (Figure 2C). The resultant cytokine production (IL-10, TGF-β, NO, HMGB-1, IFN-γ) by DC_Grp78_ also did not alter when compared with immature cells (Figure 2D), as well as gene transcription phenotyping (Cox-2, ARG-1, iNOS, TGF-β, and TNF-α, Figure 2E).

As an example of tDC, immature DCs constitutively express low levels of costimulatory molecules and adhesion molecules, as well as weak antigen presentation capacity but strong endocytic activity (18). This is consistent with our studies. The findings of this DC_Grp78_ induction protocols indicated that Grp78 induced a specific tolerogenic cell phenotype comparable to immature DCs.

### DC_Grp78_ Retained a Tolerogenic Signature after Proinflammatory Stimulation by LPS

**Figure 3:**
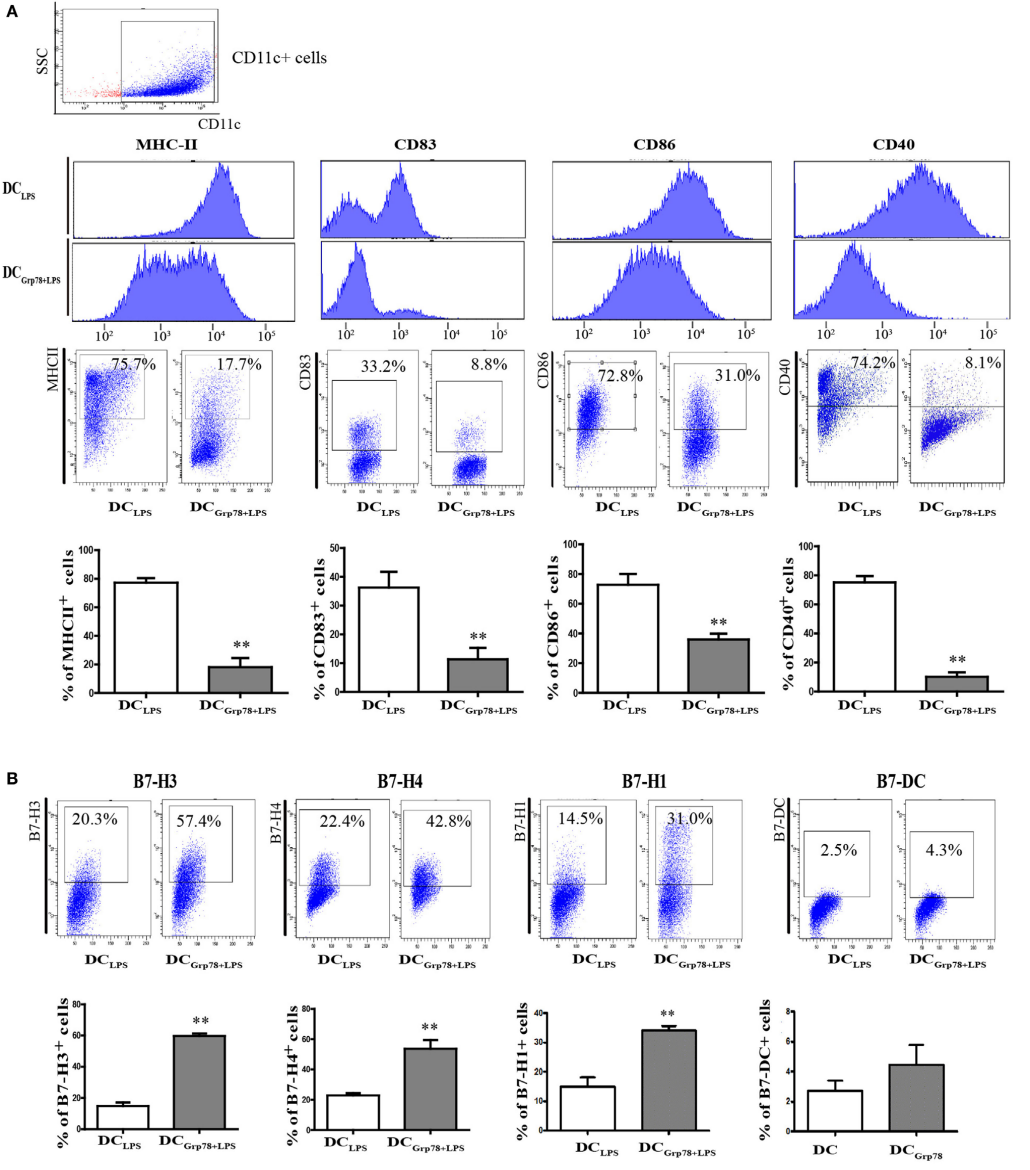

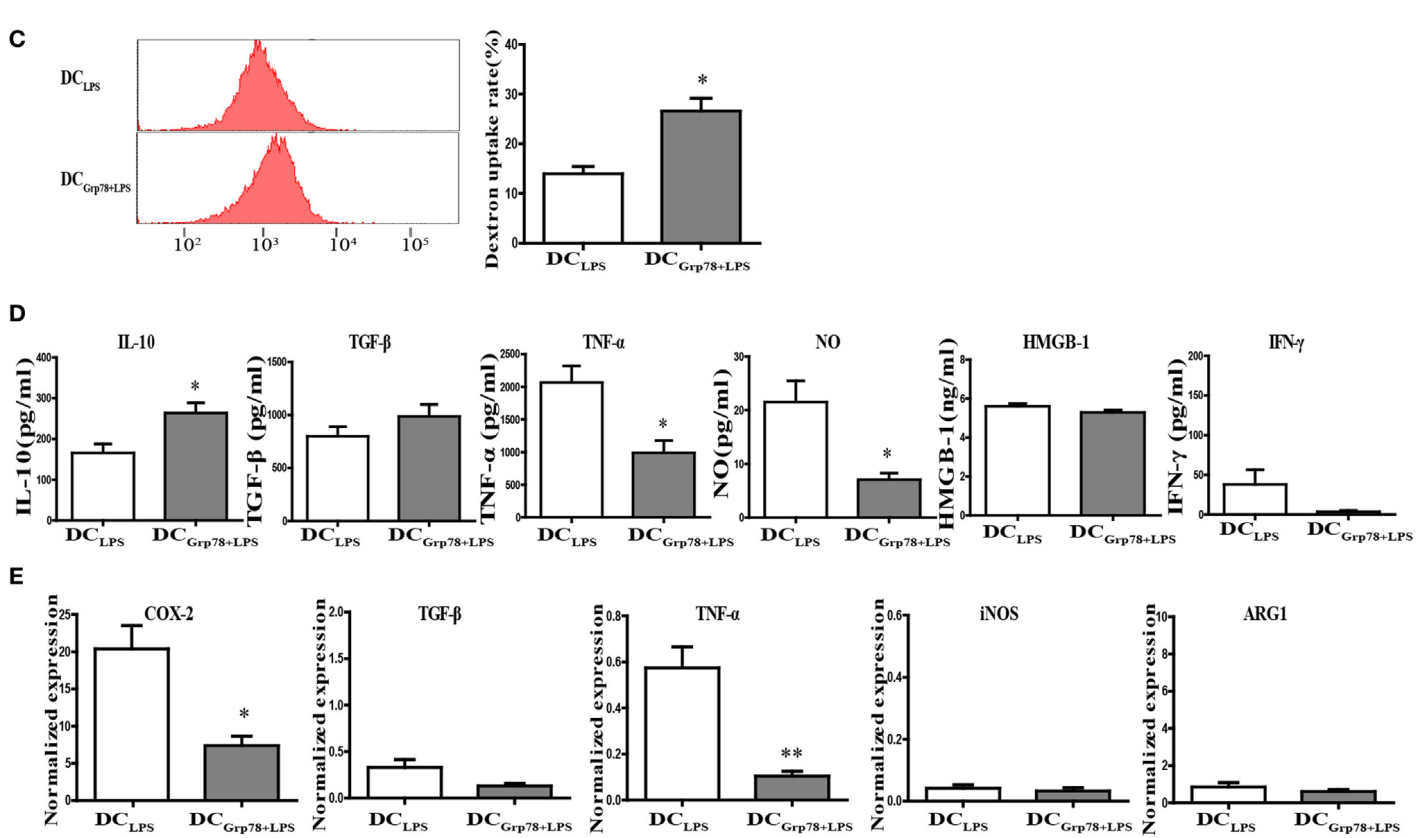
**Grp78-treated CD11c^+^ cells retained a regulatory signature after proinflammatory stimulation by LPS *in vitro***. LPS was added for the final 18–24 h during cell culture to generate DC_Grp78+LPS_ or DC_LPS_. **(A)** Flow cytometry analysis of MHC-II, CD83, CD80, and CD40 expression on DC_Grp78+LPS_ and DC_LPS_ cells. Mean fluorescence intensity and (upper), representative flow cytometric dot plots (middle), and percentage of positive cells (lower) were depicted. **(B)** Percentage of B7-H3/B7-H4/B7-H1/B7-DC cells in DC_Grp78+LPS_ and DC_LPS_ cells. **(C)** Endocytosis assessed by 1 h incubation with dextran-rhodamine. MFI (left) and percentage of rhodamine positive cells (right) were depicted. **(D)** Cell culture supernatants were analyzed for IL-10, TGF-β, TNF-α, NO, HMGB-1, and IFN-γ by ELISA. **(E)** Gene expression of COX-2, TGF-β, TNF-α, iNOS, and ARG-1 assessed by qPCR. All results were representative of three independent experiments. Error bars were presented in SEM. *P < 0.05 vs. DC_LPS_, **P < 0.01 vs. DC_LPS_.

During inflammation or infection, immature DCs undergo final maturation and become activated to induce immunity (16, 18), and no longer keep their tolerogenic properties. How to stabilize the immature DC phenotype is important for inducing tolerance in various autoimmune diseases. To assess the fate of DC_Grp78_ phenotypes after proinflammatory stimulation, DC_Grp78_ cells were stimulated with LPS for 18–24 h. Results manifested that, upon LPS stimuli, DC_Grp78_ cells significantly expressed lower levels of MHC-II, CD83, CD86, and CD40 (Figure 3A). And, the frequencies of B7-H3^+^/B7-H4^+^/B7-H1^+^ cells were not reduced and still significantly higher relative to DC_LPS_ control (Figure 3B). Furthermore, DC_Grp78+LPS_ cells retained their endocytic activity, but DC_LPS_ cells lost this activity evidently (Figure 3C). In terms of cytokine secretion, DC_Grp78_ cells produced comparably higher levels of anti-inflammatory IL-10 and lower levels of proinflammatory TNF-α and NO when stimulated with LPS (Figure 3D). LPS is a strong inducer of cyclooxygenase (COX-2) (21), which is responsible for the elevated production of PGs during inflammatory processes. Our observations supported this result, and in agreement with tolerogenic signature of DC_Grp78_ cells, level of inducible form of COX-2 in these cells was not elevated and still significantly lower compared to DC_LPS_ control (Figure 3E). These data indicated that DC_Grp78_ cells retained a tolerogenic signature and did not adopt a mature DC phenotype upon exposure to LPS.

### Grp78-Treated CD11c^+^ Cells Suppressed T-Cell Proliferation *In Vitro*

**Figure 4:**
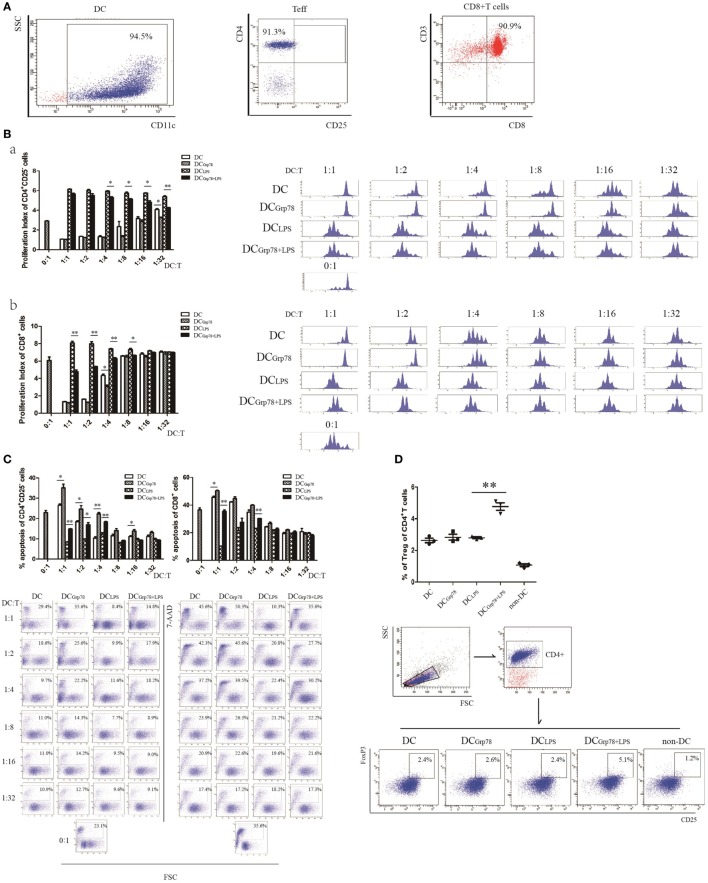
**Grp78-treated cells could suppress T-cell proliferation and induce formation of Tregs *in vitro***. **(A)** CD11c^+^ cells and CD4^+^CD25^−^, CD8^+^ T cells from splenocytes were isolated using Miltenyi MACS. The purity of CD11c^+^ cells (left), CD4^+^CD25^−^ Teff (median), and CD8^+^ T (right) cells were tested. **(B)** CFSE-stained T cells (2 × 10^5^) were cocultured with αCD3 (0.3 μg/ml) and DCs at titration indicated, for 72 h. CFSE dilution of CD4^+^CD25^−^ Teff (a) and CD8^+^ T cells (b) were analyzed. Proliferation index (left) and representative flow cytometric histograms (right) were depicted. **(C)** 7-AAD-stained CD4^+^CD25^−^ Teff (left) and CD8^+^ T (right) cells were cocultured with CD11c^+^ cells, as described above. 7-AAD^+^ cells were analyzed. Percentage of apoptosis (upper) and representative images (lower) were depicted. **(D)** CD4^+^CD25^−^ cells were cocultured with CD11c^+^ cells as described. Cells were gated for CD4^+^. CD25^+^FoxP3^+^ expressing cells were analyzed. Percentage of CD25^+^FoxP3^+^ cells (upper) and representative flow cytometric dot plots (lower) were depicted. The results were representative of three independent experiments. Statistical comparison was conducted as indicated. Error bars were presented in SEM. *P < 0.05, **P < 0.01.

Given the fact that a simple phenotypic determination of maturation markers does not guarantee a tolerogenic function and that a set of functional determinations is mandatory, an *in vitro* suppression assay was used, using anti-CD3 splenocyte activation, to clearly define tolerogenic functional phenotype of Grp78-treated cells. CD11c^+^ cells and CD4^+^CD25^−^, CD8^+^ T cells from splenocytes were enriched using Miltenyi MACS. The purity of all cell subsets was over 90% (Figure 4A). The frequency of proliferating cells was determined by the percentage of CFSE dilution. Results showed that CD4^+^CD25^−^, CD8^+^ T cells cocultured with DC_Grp78+LPS_ cells proliferated less than those with DC_LPS_ cells (Figure 4B). These results indicated that Grp78 had a role in the regulatory phenotype of CD11c^+^ cells to suppress T-cell proliferation. To elucidate the mechanism of their suppressive action, the ability of Grp78-treated cells to induce T cells’ apoptosis and to generate Tregs were assessed *in vitro*. In accordance with CFSE proliferation assay, compared with immature DCs, DC_Grp78_ treatment increased the percentage of apoptotic T cells even at a low (1:16) DC:T titration. And, T cells cocultured with DC_Grp78+LPS_ cells underwent more apoptosis than those with DC_LPS_ (Figure 4C). The increment of apoptosis could result from the elevation of B7-H1 in DC_Grp78_ and DC_Grp78+LPS_ (Figures 2B and 3B). And, Grp78 pretreatment of DC_LPS_ led to an increase in the percentage of CD4^+^CD25^+^FoxP3^+^ Tregs induced from CD25^+^-depleted CD4^+^ splenocytes (Figure 4D). Data meant that the regulation of T cell activities by Grp78-treated DCs could result from increased T-cell deletion and expansion of Tregs.

### Grp78-Treated CD11c^+^ Cells Kept Its Regulatory Function *Ex Vivo*

**Figure 5:**
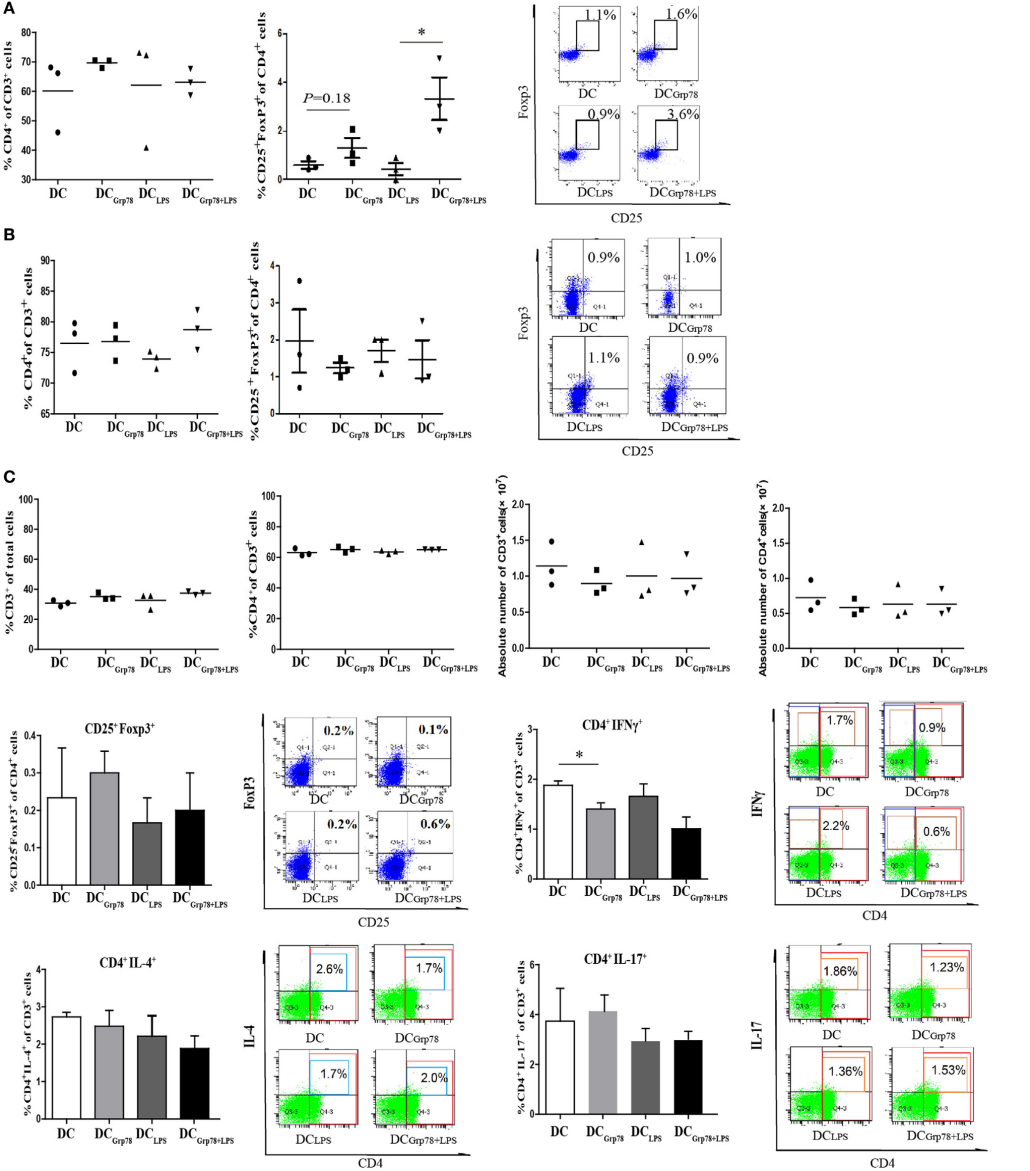

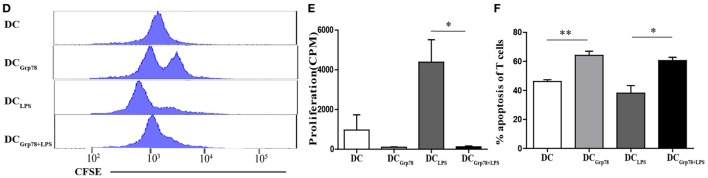
**Grp78-treated CD11c^+^ cells kept regulatory function *ex vivo***. NIT lysate-pulsed cells (5 × 10^6^) were injected into 6- to 8-week-old BALB/c mice every 7 days for three times. Mice were sacrificed 1 week after final injection, and PLNs, blood, and spleen were dissected. Lymphocyte subsets from PLNs **(A)** and blood **(B)** were analyzed by flow cytometry for percent values. Cells were gated for CD3^+^. Percentage of CD4^+^ in CD3^+^ cells (left), percentage of CD25^+^FoxP3^+^ in CD4^+^ cells (median), and representative flow cytometric dot plots (right) were depicted. **(C)** Splenocytes were analyzed by flow cytometry for percent and absolute values. Frequencies and representative flow cytometric dot plots of Treg (CD4^+^CD25^+^FoxP3^+^), Th1 (CD4^+^IFNγ^+^), Th2 (CD4^+^IL-4^+^), and Th17 (CD4^+^IL-17^+^) cells were also indicated. **(D,E)** Splenocytes were reprimed with mitomycin-treated NITs for 5 days **(D)** or 3 days **(E)**. Then, cell proliferation was assayed by CFSE dilution **(D)** or ^3^H-TdR incorporation **(E)**. **(F)** Splenocytes were reprimed by mitomycin-treated NITs for 7 days and then subjected to CFSE staining. After coculture with NITs for 12 h, cell apoptosis in CFSE^+^CD3^+^ T cells was assayed. All results were representative of three independent experiments. Error bars are presented in SEM. *P < 0.05, **P < 0.01.

To address the local *in vivo* mechanism of action of Grp78-treated CD11c^+^ cells, BALB/c mice were injected with NIT lysate-pulsed CD11c^+^ cells for three times. PLNs, blood, and spleen were dissected out 1 week after final injection. Although the relative numbers (percentage of cells or absolute numbers) of CD4^+^ T cells and Treg (CD4^+^CD25^+^FoxP3^+^), Th1 (CD4^+^IFNγ^+^), Th2 (CD4^+^IL-4^+^), and Th17 (CD4^+^IL-17^+^) cells in blood or spleen did not alter among different CD11c^+^ cells treatment (Figures 5A–C), in PLNs, there was a statistical difference of frequency of CD4^+^CD25^+^FoxP3^+^ Tregs in DC_Grp78+LPS_ treated mice compared with DC_LPS_ group (Figure 5A). When splenocytes were reprimed by NITs, antigen-specific proliferation was determined by CFSE dilution (Figure 5D) and ^3^H-TdR incorporation assay (Figure 5E). Splenocytes from DC_Grp78+LPS_ treated mice proliferated less than those from DC_LPS_ treated mice, clearly indicated immunomodulation of lymphocytes by Grp78-treated CD11c^+^ cells *ex vivo*. And, the immunomodulation may partly result from the increased apoptosis of lymphocytes in Grp78-DCs treated groups (Figure 5F).

## Discussion

Dendritic cells exist in two basic functional states: immature DCs induce tolerance to self, whereas mature DCs induce immunity to foreign antigens. When receiving maturation signals in the form of microbial patterns, danger signals, or inflammatory cytokines, immature DCs lose their tolerogenic functions and transit to mature DCs that can prime T cells (18–20). Stabilization of the immature DC phenotype can contribute to induce durable tolerance to transplanted allogeneic grafts and suppress the development of autoimmune diseases. Attempts have been made *in vivo* and *in vitro*. For instances, IL-10 was used to modulated DCs from peripheral blood to induce antigen-specific anergy in CD4^+^ and CD8^+^ T cells (22). Immature DCs can also response to 1,25-dihydroxyvitamin D3, dexamethasone (23), and rapamycin (24) to be matured towards a Treg-inducing or tolerogenic state. Li et al. (25) even reported that stimulation of DC with IFN-γ and CD40L resulted in rapid induction of IDO transcription and recapitulated the *in vivo* switch from immunogenic to tolerogenic activity.

In this research, it was found that myeloid APCs could bind with recombinant murine Grp78, and *in vitro* phenotypic analyses revealed that the Grp78 treatment induced a tolerogenic cell phenotype comparable to immature DCs. When assessing the fate of DC_Grp78_ phenotypes upon maturation signals, DC_Grp78_ cells were cultured in the presence or absence of LPS as a maturation stimulus, which have been shown that LPS-matured DCs acquire an enhanced ability to stimulate specific T cells and to block regulatory T-cell activity (26). Data indicated that after stimulation by strong TLR4 ligand, DC_Grp78_ cells were comparatively resistant to maturation and did not adopt a mature DC phenotype. Among these phenotypic molecules, MHC-II showed significant decrease not only in DC_Grp78_ but also in DC_Grp78+LPS_. It needs further investigation for how Grp78 signaling affects the expression of MHC-II.

Another functional property of tDCs is their decreased T cell-stimulatory capability (27). The following *in vitro* cell proliferation assay manifested that Grp78-treated DCs had potentials for reducing T proliferations. It has been described that tDCs regulate T cell activities through several pathways, including clonal T cell depletion or exhaustion by a mechanism dependent on the induction of apoptosis, anergy, deviation of Th differentiation, or generation of Tregs (28, 29). To deduce which mechanisms that Grp78-treated myeloid APCs might have exerted, the possibility of apoptosis induction were evaluated. We did find significant differences in cell death by stimulated T cells, indicating that this mechanism was acting in our Grp78-treated cellular products. For the generation of Tregs, Grp78-DC-primed T cells exhibited reduced proliferation along with a concomitant expansion of CD4^+^CD25^+^FoxP3^+^cells. But, it is unclear whether CD11c^+^ cells only induce Treg or also contribute to the expansion of natural Treg. The *in vitro* immunomodulatory property of Grp78-treated CD11c^+^ cells was confirmed by *ex vivo* T cell proliferation. Functional analyses revealed that these Grp78-treated CD11c^+^ cells could modulate the *ex vivo* antigen-specific activation of splenocytes. It can be suggested that in the proinflammatory environment, the transferred Grp78-treated CD11c^+^ cells retained the tolerogenic signature and deactivated infiltrated T cells through induction of lymphocyte apoptosis and Treg formation. These anti-inflammatory effects may have been in response to the expression of low levels of CD86 and of high levels of co-inhibitory molecules on Grp78-treated CD11c^+^ cells and is consistent with previous reports that CD28 costimulation negatively regulates the generation of Tregs (30) and B7-H3/B7-H4/B7-H1 are linked to limited T cell proliferation (31). Given the fact that during an immune response DCs can be exposed to a variety of stimuli in different combinations (32), future work need to be done to test whether DC_Grp78_ cells could retain tolerogenic signature once in the prolinflammatory environment, like CpG, TNF, IL-1β, IFNs, anti-CD40, and so on.

In regard to cytokine profile, Grp78-treated CD11c^+^ cells increased the production of IL-10, while showed no significant change for the expression of TGF-β. Zhang et al. (33) reported that GRP78 overexpression facilitated the expression and secretion of TGF-β1 in colon cancer cells. This discrepancy could be due to the different cell types used and Grp78 functioning in different ways when it resides in different cell compartments.

A widely used protocol involves the culture of murine BM cells with GM-CSF to generate BMDCs. BMDCs express CD11c and MHC-II molecules and share with DCs isolated from tissues the ability to present exogenous antigens to T cells and to respond to microbial stimuli by undergoing maturation (34). GM-CSF plus IL-4 is a widely used protocol to culture murine BM cells to generate BMDCs (21, 35–37). CD11c^+^ cells obtained in this manner constitute pure DC populations although the cellular heterogeneity has been appreciated. In the paper, this protocol was applied to generate CD11c^+^ myeloid APCs. The purity of CD11c^+^ was over 80%. Helft et al. reported that mouse BM cultured in GM-CSF generates DCs and macrophages, which both express CD11c and MHC class II. IL-4 does not prevent the development of monocyte-derived macrophages. And, the numbers of GM-CSF-macrophage needed to induce specific CTL response is 10-fold higher than those of GM-CSF-DC (34). Hence, it is worth of our further studies to refine *in vitro* DC culture systems to gain a better understanding of the function of DCs and macrophages and has implications for the design of cell-based therapies for use in humans.

In summary, we verified that Grp78 regulates the maturation of myeloid APCs to produce a tolerogenic phenotype, and such phenotype is stable upon LPS stimulation. These Grp78-treated myeloid APCs could reduce T proliferations in a way that induce T cell apoptosis and generate Tregs *in vitro* and *ex vivo*. The above work supports that Grp78 is an immunomodulatory molecule. It may thus contribute to induce durable tolerance to be of potential therapeutic benefit in autoimmune diseases such as type I diabetes.

## Author Contributions

MY and FZ performed all the described experiments except for Grp78 binding. KQ provided Grp78-binding analysis. MW and HL prepared the murine recombinant Grp78. HZ cultured the BMDCs. PL, QN, and GS provided experimental help and design. PL wrote the manuscript.

## Conflict of Interest Statement

The authors declare that the research was conducted in the absence of any commercial or financial relationships that could be construed as a potential conflict of interest. The reviewer AJ and handling Editor declared their shared affiliation, and the handling Editor states that the process nevertheless met the standards of a fair and objective review.
